# Clinical Outcomes after Treatment of Periodontal Intrabony Defects with Nanocrystalline Hydroxyapatite (Ostim) or Enamel Matrix Derivatives (Emdogain): A Randomized Controlled Clinical Trial

**DOI:** 10.1155/2014/786353

**Published:** 2014-02-09

**Authors:** Elyan Al Machot, Thomas Hoffmann, Katrin Lorenz, Ihssan Khalili, Barbara Noack

**Affiliations:** Department of Periodontology, Medical Faculty Carl Gustav Carus, TU Dresden, Fetscherstraße 74, 01307 Dresden, Germany

## Abstract

*Introduction*. Periodontitis is an inflammatory process in response to dental biofilm and leads to periodontal tissue destruction. The aim of this study was the comparison of outcomes using either an enamel matrix derivative (EMD) or a nanocrystalline hydroxyapatite (NHA) in regenerative periodontal therapy after 6 and 12 months. *Methods*. Using a parallel group, prospective randomized study design, we enrolled 19 patients in each group. The primary outcome was bone fill after 12 months. Attachment gain, probing pocket depth (PPD) reduction, and recession were secondary variables. Additionally, early wound healing and adverse events were assessed. Data analysis included test of noninferiority of NHA group (test) compared to EMD group (reference) in bone fill. Differences in means of secondary variables were compared by paired *t*-test, frequency data by exact **χ** ^2^ test. *Results*. Both groups showed significant bone fill, reduction of PPD, increase in recession, and gain of attachment after 6 and 12 months. No significant differences between groups were found at any time point. Adverse events were comparable between both groups with a tendency of more complaints in the NHA group. *Conclusion*. The clinical outcomes were similar in both groups. EMD could have some advantage compared to NHA regarding patients comfort and adverse events. The trial is registered with ClinicalTrials.gov NCT00757159.

## 1. Introduction

Periodontal disease is an inflammatory response that, in the absence of systematic periodontal treatment, leads to periodontal tissue loss. Conventional surgical approaches, such as open flap debridement, offer only limited potential in reconstituting components of periodontal tissues.

Preclinical and clinical studies have approved the role of enamel matrix derivatives (EMD) in conjunction with open flap to stimulate periodontal regeneration and to reconstitute the lost periodontal structures (i.e., the new formation of root cementum, periodontal ligament, and alveolar bone) [1–6]. Although EMD has demonstrated the ability to promote angiogenesis and osteogenesis both in vitro and in vivo, the specific elements within the EMD compound responsible for these effects remain unknown [7].

Systematic reviews [8, 9] reported that the treatment of deep intrabony defects with alloplastic grafts, which are synthetic, inorganic, biocompatible bone graft substitutes, provided additional gain of clinical attachment level (CAL) and additional probing pocket depths (PPD) reductions compared to open flap debridement alone. A ready-to-use nanocrystalline hydroxyapatite (NHA) paste, available under the brand name Ostim (Heraeus Kulzer, Hanau, Germany), containing about 35% nanoscopic apatite particles in aqueous dispersion, is currently available for use in orthopaedic trauma surgery and has been recommended for augmentation procedures in osseous defects [10–13].

In dentistry, experimental studies have demonstrated that NHA paste is a stimulator of cell proliferation, possibly contributing to the main processes of periodontal tissue regeneration [14]. Clinical and biomolecular observations evidenced that NHA improves alveolar socket healing, increasing angiogenesis and epithelialization, as well as osteogenesis through increasing the synthesis of proosteogenic factors as bone morphogenetics protein BMP-4, BMP-7, alkaline phosphatase, and osteocalcin [15]. The regenerative treatment of intrabony periodontal defects with a NHA paste led after 6 months to significantly improved clinical outcomes when compared with papilla preservation flap surgery alone [16].

Previous in vitro study evaluated the role of soluble or coated NHA paste and EMD on proliferation, adhesion, and migration of periodontal ligament fibroblasts (PDLs) [17]. This study presented evidence that NHA paste and EMD mediate their beneficial effects on periodontal tissues via two different modes of action and therefore have different molecular characteristics. EMD exhibited a pronounced chemotactic effect once applied to solution, and NHA supported cellular adhesion in its solid state, providing a basis for PDL fibroblasts to settle down. A current in vitro study did show that the initial root surface colonization by human periodontal ligament fibroblasts may be enhanced by the application of EMD compared to the treatment with NHA paste [18]. However so far, no controlled clinical studies have evaluated the healing of peridontal bone defects following regenerative treatment with NHA compared to the treatment with EMD.

Therefore, the aim of this study was to compare the clinical outcomes following papilla preservation flap (PPF) surgery performed by dental practitioners using either an EMD or a NHA paste in wide (>2 mm) and deep (≥4 mm) one- and two-wall intrabony defects 6 and 12 months after treatment. It was hypothesised that both therapy modalities have at least comparable outcomes. Therefore, specific aims were to analyze (i) clinical measurements of periodontal regeneration, (ii) early wound healing, and (iii) patient's perceptions and adverse effects of both treatment modalities.

## 2. Materials and Methods

### 2.1. Study Design and Sample

A parallel group, randomized, prospective, and controlled clinical trial was designed to evaluate the clinical outcomes 6 and 12 months following PPF surgery using either EMD or a synthetic bone graft (NHA paste) in wide intrabony defects.

Periodontitis patients referred to the Department of Periodontology, Dental School, University of Technology, Dresden, Germany, for periodontitis therapy were screened for inclusion. Thirty-eight generally healthy patients (18 females and 20 males; aged from 30 to 65 years) were selected. Study inclusion criteria were as follows: (1) no systemic diseases that could influence periodontal wound healing or periodontal progression (e.g., diabetes mellitus, rheumatoid arthritis, and cancer); (2) no use of antibiotics during the previous 6 months; (3) good oral hygiene with a full-mouth plaque score ≤30% before inclusion; (4) nonsmokers, former smokers (defined as nonsmokers for at least 5 years), and occasional smokers (based on self-reported cigarette consumption of 1–30 cigarettes/month at maximum); (5) severe periodontitis previously treated by oral hygiene instructions and subgingival scaling and root planing, at least 6 weeks prior to the start of the study; (6) presence of a single intrabony defect with more than 2 mm radiographic width and at least 4 mm depth (depth, type of the intrabony defect, and furcation involvement were evaluated during screening but had to be confirmed during surgery). Furthermore, patients were not included if they suffered from unstable or life-threatening conditions, if they were pregnant or lactating, if caries or untreated endodontic problems were present, if the intrabony defects extended into a furcation area, or if 3-wall defects existed.

All patients received a description of the study and signed a written informed consent form. The study was performed in compliance with Good Clinical Practice and the Declaration of Helsinki last revised in Edinburgh 2000. The study protocol was reviewed and approved by the Ethics Committee in Dresden, Faculty of Medicine Carl Gustav Carus, TU Dresden, Germany (reference number EK145062008).

### 2.2. Study Variables and Data Collection Methods

Clinical variables were evaluated at baseline, 6, and 12 months after regenerative therapy. Evaluated clinical periodontal variables included bone level, PPD, relative attachment level (RAL), and relative gingival recession (RGR) at one treated tooth per patient. Additionally, full-mouth plaque score [19] was assessed. All parameters were recorded at six sites per tooth (distobuccal, mediobuccal, mesiobuccal, distolingual, mediolingual, and mesiolingual). Bone level, PPD, RAL, and RGR were assessed with a standard periodontal manual probe (PCP-UNC 15, Hu-Friedy, Leimen, Germany) using an acrylic customized stent with markings at six fixed reference points. The clinical bone level was measured by bone sounding following local anesthesia and was defined as the distance between the margin of the stent and the bottom of the bone defect. RAL and RGR were evaluated by measuring the distance between the margin of the stent and the bottom of the clinical pocket and gingival margin, respectively. PPD was recorded as the distance between the bottom of the clinical pocket and gingival margin. The primary outcome was mean bone fill calculated as the difference between bone level at baseline and bone level at 12 months after surgery.

**Figure 1:**
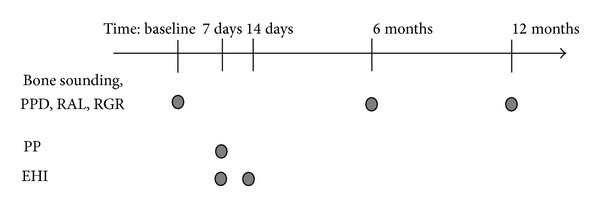
Study design. PPD: probing pocket depth; RAL: relative attachment level; RGR: relative gingival recession; PP: patient perception; EHI: early wound-healing index.

Seven days after surgery, patients were asked about the degree (severe, moderate, mild, and none) of pain, bleeding, and swelling during the first week after treatment using a questionnaire. Postoperative healing was assessed by the “early wound-healing index” (EHI) at 7 and 14 days after surgery [20]. EHI differs between 5 degrees of healing: (1) complete flap closure—no fibrin line in the interproximal area; (2) complete flap closure—fine fibrin line in the interproximal area; (3) complete flap closure—fibrin clot in the interproximal area; (4) incomplete flap closure—partial necrosis of the interproximal tissue; (5) incomplete flap closure—complete necrosis of the interproximal tissue. Intraoral radiographs were taken before surgery and at 12 months using the long cone paralleling technique. The study design is shown as a flow chart in Figure 1.

Two blinded examiners were responsible for all study measurements. A calibration exercise was performed to obtain acceptable intraexaminer reproducibility of the first examiner for the measurement of PPD and RAL. The sites measured were comparable to the sites to be measured in the study. Repeated measurements were performed at least one hour later. Calibration was accepted if measurements before and after 1 hour were identical at >90% of sites. The second examiner recorded the morphology and measurements of the intrabony defects during surgery (for details see below).

### 2.3. Treatment Protocol

**Figure 2:**
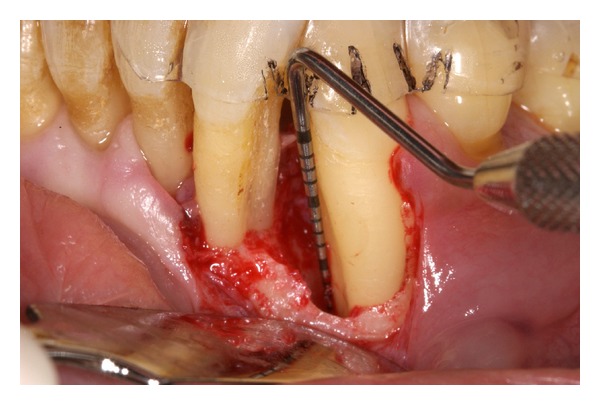
Intraoperative view of the intrabony defect after debridement. All clinical parameters were assessed at six sites per tooth with a standard periodontal manual probe (PCP-UNC 15, Hu-Friedy, Leimen, Germany) using an acrylic customized stent with markings at six fixed reference points. The same stent was used for all clinical measurements before and after surgery.

Dental practitioners with different surgical experience have performed the surgical procedures under supervision of periodontal specialists, as part of their master degree program (Master of Science in Periodontology and Implant Therapy, German Society of Periodontology and Dresden International University) in the Department of Periodontology, Dental School, TU Dresden. The supervisors determined the surgical procedure, flap extent and design, which was in general identical for both groups. Following local anesthesia, a full thickness (mucoperiosteal) access flap was elevated using the modified papilla preservation technique [21]. Infected granulation tissue and any remaining subgingival calculus were removed. Careful scaling and root planing was carried out with hand instruments and oscillating scalers. After complete debridement of the surgical site, the second examiner screened the morphology of the defect and performed following measurements using a manual probe (PCP-UNC 15, Hu-Friedy, Leimen, Germany): (1) intraoperative bone level (distance from stent to bottom of the defect); (2) defect depth (distance from bone crest to bottom of bone defect); (3) defect width (distance between root surface and bone crest); and (4) determination of the defect type (1-wall, 2-wall, combined 1- and 2-wall, or circumferential). Defects with an intrabony component of ≤2 mm width and <4 mm depth were not included in the study (Figure 2). The defects were randomly assigned to a treatment procedure by the flip of a coin by the supervisor. In the EMD group, the exposed root surface was conditioned with EDTA gel (PrefGel, Straumann, Basel, Switzerland) for 2 min. After thoroughly rinsing with saline and making sure that no blood or saliva contaminated the root surface, EMD (Straumann Emdogain, Straumann, Basel, Switzerland) was then directly applied to the exposed root surface. In the NHA group, bleeding into defects was reduced to a minimum before filling with NHA paste (Ostim, Heraeus Kulzer, Hanau, Germany). NHA was then gently packed into the defects and filled up to the highest level of the defect walls. In both groups, flaps were subsequently replaced. Great care was taken to obtain complete closure in the area of the interdental papilla above the treated defect without any tension. Monofilament nonabsorbable 5-0 and 6-0 suturing material was used.

All patients were advised to rinse the mouth twice a day with 0.2% chlorhexidine digluconate solution for the first 4 postoperative weeks. Mechanical oral hygiene was not allowed in the surgical areas during this time; however, a gently cleaning of occlusal areas with a supersoft toothbrush was allowed. No medications were prescribed postoperatively. After 10–14 days, sutures were removed. After 7 days, 14 days, and 6 weeks, all patients underwent gentle supragingival professional tooth cleaning and reinforcement of oral hygiene if necessary. A maintenance program was set up for all patients at 3 and 6, 9, and 12 months. However, no subgingival instrumentation or probing was performed at the surgical site during the first 6 months after surgery. At any visit, adverse events including postsurgical complications were recorded.

### 2.4. Data Management and Statistical Analysis

For data processing and statistical evaluation, appropriate validated software was used (SPSS software package, version 17, SPSS, Chicago, IL, USA). Descriptive summary statistics were computed for all parameters documented. All descriptions were done separately for treatment groups and visits. Group mean ± SD were calculated for each clinical parameter. The deepest site per tooth was included in the calculations.

The primary outcome variable in this clinical study was the change in bone level (bone fill) comparing the effect of EMD (reference treatment) versus NHA (test treatment) between baseline and 12 months after surgery. The following hypothesis was tested: the change in bone level between baseline and 12 months after surgery is not worse after defect filling with NHA compared to EMD. Noninferiority margin of the test treatment was defined by a clinically irrelevant difference up to approximately 30% defect fill. Assuming an effect size is 1, that is, ratio of tolerance limit/standard deviation, a sample size of 18 patients was estimated to have 95% power to test for noninferiority with a significance level *α* = 0.1 (one tailed). 19 patients were treated in each group of this study. Confirmative statistical testing of the noninferiority hypothesis was performed by computing a 95% confidence interval (CI) for the mean change in bone fill after 12 months in the NHA group and comparing it with the mean change in bone fill after 12 months in the EMD group minus 30%.

Bone fill after 6 months, clinical attachment gain, reduction of PPD, and changes in gingival recession at 6 and 12 months were considered secondary outcome variables. In addition, early wound healing and patient's perceptions at 7 and 14 days after surgery were evaluated. Between-group differences at baseline and 6 and 12 months after surgery (EMD versus NHA) were tested using the unpaired *t*-test. The Mann-Whitney *U* test was used in case of ordinal data. Frequency data were compared by the **χ** ^2^ test, computing exact *P* values, or Fisher's exact test. The paired *t*-test was utilized to evaluate differences between baseline and followup within each group. The significance level was set at *P* < 0.05 for all group comparisons.

## 3. Results

### 3.1. Patient and Defect Characteristics

**Figure 3:**
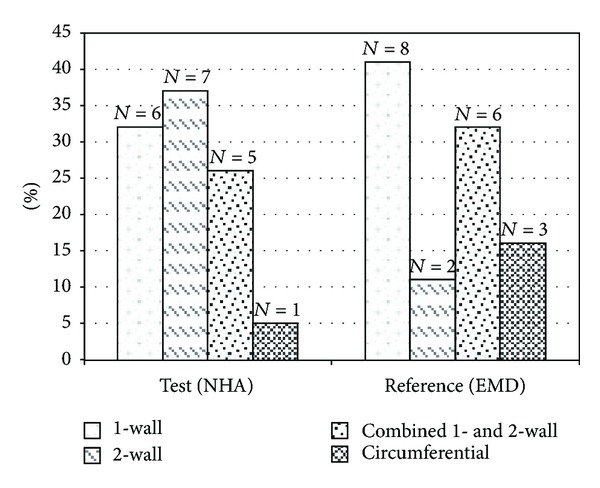
Distribution (%) of defect types as assessed during surgical intervention. NHA: nanocrystalline hydroxyapatite; EMD: enamel matrix derivate.

After 12 months, a total of 38 patients, 19 in each group, completed the follow-up period. No data were missing. No statistically significant differences were found between the groups for any of the investigated parameters at baseline (Table 1). The population consisted of 18 females and 20 males; aged from 35 to 65 years. The means of defect depth measured from bone crest to bottom of the debrided bone defect were 6.5 mm (±1.6) and 5.6 mm (±1.8) for the NHA and EMD groups, respectively. Defect width reached a mean of 3.4 mm (±0.7) in the NHA group and 3.2 mm (±0.7) in the EMD group (Table 1). There were no significant differences for any of the defect characteristics in both groups at baseline. Distributions of defect types are displayed in Figure 3.

### 3.2. Clinical Outcomes

The comparison of clinical measurements (bone level, PPD, RAL, and RGR) at baseline, 6 months, and 12 months after surgery is summarized in Table 2. Compared to the baseline data, the NHA and EMD groups showed statistically significant reduction of PPD, increase in recession, and gain of attachment after 6 and 12 months (*P* < 0.001). Both treatment modalities led to significant bone fill measured by bone sounding after 6 and 12 months compared to baseline. In the NHA group, mean bone fill of 1.7 mm (95% CI (0.8–2.5), *P* = 0.001, paired *t*-test) and 1.6 mm (95% CI (1.0–2.2), *P* < 0.001, paired *t*-test) was observed after 6 and 12 months, respectively. In the EMD group, mean defect fill was 1.8 mm (95% CI (1.1–2.3), *P* < 0.001, paired *t*-test) after 6 month and 1.6 mm (95% CI (1.0–2.3), *P* < 0.001, paired *t*-test) after 12 months. Both 95% CI of the NHA group included the means of the EMD group minus 30% (1.2 mm; 95% CI (0.7–1.6) and 1.1 mm; 95% CI (0.7–1.6)). Thus, noninferiority of the test treatment with NHA can be claimed taking into account the noninferiority definition of a clinically irrelevant difference up to approximately 30% defect fill. Between groups, no significant differences of secondary clinical outcomes (PPD, RAL, and RGR) were found for any of the variables at 6 and 12 months (Table 2). Furthermore, a comparison of mean differences in clinical measurements (bone level, PPD, RAL, and RGR) as calculated after 12 months with the mean differences as calculated after 6 months showed no statistically significant changes in both groups (Table 2, *P* > 0.05, paired *t*-test).

**Figure 4:**
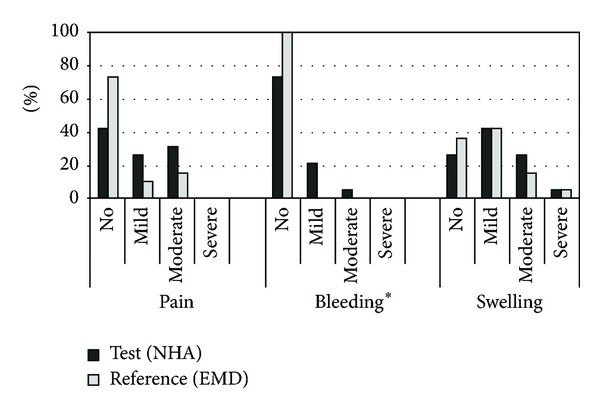
Distribution (%) of patient perceptions 1 week after treatment. *P = 0.046; exact **χ** ^2^ test. EMD: enamel matrix derivate; NHA: nanocrystalline hydroxyapatite.

**Figure 5:**
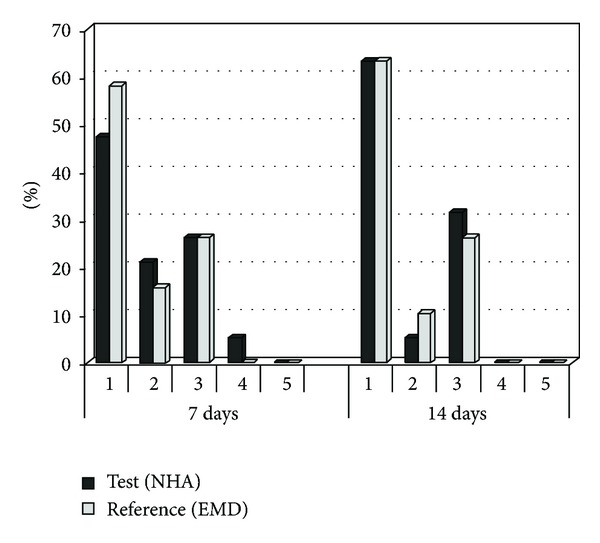
Distribution (%) of early wound-healing index at the treated sites, at 1 and 2 weeks after treatment. No significant differences between groups (7 days: *P* = 0.511; 14 days: *P* = 0.904, Mann-Whitney *U* test) EMD: enamel matrix derivate; NHA: nanocrystalline hydroxyapatite.

The mean full-mouth plaque scores ranged between 9.6% and 28.4% at all evaluation time points with no significant differences between groups at any visit. However, mean plaque values after 3, 6, 9, and 12 months were increased in both groups compared to baseline; the changes were statistically significant (*P* < 0.05; Table 3). The results of the self-reported postoperative healing events within the first 7 days are summarized in Figure 4. The results were comparable between both groups with a tendency of more complaints in the NHA group compared to the EMD group (moderate pain: 32% versus 16%, bleeding: 26% versus 0%, moderate and severe swelling: 32% versus 21%, resp.). The difference in frequency distribution of bleeding was significant (*P* = 0.046; exact **χ** ^2^ test). The percentage of patients with EHI scores 1–5 at 1 and 2 weeks after treatment is demonstrated in Figure 5. The comparison of the results of EHI after 7 and 14 days showed no significant differences between groups (7 days: *P* = 0.511; 14 days: *P* = 0.904, Mann-Whitney *U* test). 63% of the patients in both groups showed complete flap closure without a fibrin line in the interproximal area after two weeks (EHI score 1).

## 4. Discussion

The aim of the here presented study was to compare therapy outcomes after two different regenerative periodontitis treatment modalities. The results of this randomized-controlled trial demonstrated favourable clinical outcomes after 6 and 12 months following the application of either EMD or NHA. Both treatment modalities led to comparable results for bone gain, attachment gain, and pocket reduction at 6 and 12 months after treatment.

Different treatment modalities and materials with varying degrees of success have been used to promote periodontal wound regeneration such as bone grafts, guided tissue regeneration, EMD, growth and differentiation factors, and stem cells. In general, the application of EMD alone does not prevent the risk of a collapse of the soft tissue into wide intrabony defects. Therefore, attempts have been made to use different space-maintaining materials, such as membranes or bone replacement grafts (autogenous, allogeneic, xenogeneic, and alloplastic), alone or combined with EMD in order to promote bone formation and periodontal regeneration [6, 8, 22–24].

In the present study, clinical attachment gains and PPD reductions after 12 months noted in the group treated with EMD are comparable to previously reported data for a treatment with either EMD or a combined treatment of EMD and synthetic bone graft, which demonstrated significant improvements in PPD and CAL [25]. These clinical improvements support the existing information on the applicability of EMD in the reconstructive treatment of one- and two-wall intrabony defects [2, 20, 25–29].

The clinical outcomes in the present study following NHA application showed significant improvements after 6 and 12 months compared with baseline. These findings have been confirmed by other clinical studies which provide information on the applicability of NHA in the reconstructive treatment of one- and two-wall intrabony defects [16, 30]. After 6 months, the two randomized controlled clinical studies reported a higher clinical attachment gain of 4.4 ± 1.7 mm and 4.3 ± 1.4 mm, respectively, and PPD reductions of 3.4 ± 1.2 mm and 4.3 ± 1.1 mm, respectively, after papilla preservation flap surgery with or without the application of a NHA in intrabony periodontal defects of ≥4 mm in a split-mouth design. On the contrary, another clinical study showed that the additional use of NHA combined with an open flap procedure does not improve clinical and radiographic treatment outcomes [31]. However, NHA was used in the form of granules in contrast to paste used in the here presented study. Differences in the physicochemical and structural characteristics between paste and granules could lead to differences in the regenerative and osteoconductive properties [17]. Furthermore, the combination of NHA bone graft with a bioresorbable collagen membrane demonstrated clinical advantages beyond that achieved by open flap debridement (OFD) alone [32], although a group treated with NHA alone was not included in this study.

EMD and NHA have demonstrated the ability to promote angiogenesis and osteogenesis both in vitro and in vivo, but it is important to mention that EMD and NHA paste display different molecular characteristics and apply alternative routes to mediate their beneficial effects on periodontal tissues [17]. EMD stimulates the vascular endothelial growth factor (VEGF) production partially via transforming growth factor-*β*1 (TGF-*β*1) and fibroblast growth factor 2 (FGF-2) in human gingival fibroblasts. EMD-induced VEGF production is regulated by pathways of an extracellular signal-regulated kinase inhibitor, p38 mitogen-activated protein kinase inhibitor, and phosphatidylinositol 3-kinase (PI3K)/Akt inhibitor [33]. PDLs migration towards a NHA or EMD gradient was more efficiently mediated by soluble EMD compared with NHA. On the other hand, adhesion of PDLs to compound-coated dishes was more effectively mediated by NHA compared with EMD [17]. The authors of this in vitro study concluded that these results implicate a potential synergy between both materials and a putative beneficial effect for the wound healing of patients if applied in combination. Further studies should reveal if these findings can be transferred to a clinical setting. Another in vitro study demonstrated that the application of EMD improved the initial root surface colonization by human periodontal ligament fibroblasts compared to NHA paste [18]. Our study demonstrated comparable clinical outcomes for bone gain, attachment gain, and pocket reduction following the application of either an EMD or a NHA at 6 and 12 months after treatment. However, it remains unclear to what extent the improvement of the clinical parameters (clinical attachment gain and bone fill) following the application of NHA represents regeneration of the lost periodontal structures [34].

The primary closure of the interdental space is one of the most significant factors which can affect the outcomes after periodontal regenerative surgery [35, 36]. The present study did not find substantial healing complications in both treatment modalities assessed by EHI. Heinz and coworkers (2010) reported uneventful healing in all patients of papilla preservation flap surgery with or without the application of NHA in split-mouth design. However, they did not report how they evaluated the healing and the patient's perceptions after treatment [16]. On the other hand, in a previous clinical trial evaluating the healing of intrabony peri-implantitis defects following application of a NHA or a bovine-derived xenograft in combination with a collagen membrane, NHA showed compromised initial adhesion of the mucoperiosteal flaps in all patients [37]. The reasons for this considerable heterogeneity in clinical outcomes are numerous. They might be explained by differences in the type of included defects, surgical flap design, surgical skills, or case selection [38]. In our study, wide (>2 mm) and deep (≥4 mm) one- and two-wall intrabony defects were included. Great care was taken to achieve primary closure of the interdental area without any tension.

The handling of both materials seems to be similar in terms of simple application and patient's comfort. In the EMD group, a trend for lower rates of pain and no bleeding a week after surgery was observed. Considering the overall low event rates in both study groups, these results have to be interpreted cautiously regarding a potential advantage of EMD in terms of patient's comfort or adverse event rate. A further aspect of our study was that, in general, the surgical part of regenerative clinical studies is performed by a single periodontal specialist in order to achieve a standardised approach. However, in this study, dental practitioners with different periodontal surgical skills performed the surgical procedures under supervision of periodontists. According to our results which were comparable to other regenerative clinical studies as mentioned above, it can be concluded that the improvements of the clinical parameters after regenerative treatment with either EMD or NHA could also be achieved by dental practioners if the minimal invasive technique was used.

The small sample size in our study limits the interpretation of the observed regenerative effects in light of the defect morphology. Further studies using more subjects and histologic analysis could clarify the benefits of the application of NHA in the peridontal regenerative therapy. Future studies will have to identify the characteristics of defects that may benefit the most from either a combined or single treatment strategy involving EMD and a synthetic bone graft.

In summary, the clinical outcomes of regenerative periodontitis therapy were similar in both study groups also under conditions of a general dental praxis. A longer posttreatment observation interval may be needed to confirm the stability of the clinical outcomes observed here at 6 and 12 months. Furthermore, studies with larger sample size are needed to prove a potential advantage of EMD compared to NHA regarding patients comfort and adverse event rates.

## Figures and Tables

**Table 1:** Patient and defect characteristics at baseline.

Variable	Treatment		*P* value
	NHA group, *N* = 19	EMD group, *N* = 19	
Age (years, mean ± SD)	50.9 ± 12.9	51.8 ± 11.4	0.822*
Men/women (*n*)	12/7	8/11	0.194^†^
Smoking habits			
Nonsmoker (*n*/%)	11 (57.9)	17 (89.5)	0.063^‡^
Former/occasional smoker (*n*/%)	8 (42.1)	2 (10.5)	
PPD (mm, mean ± SD)	6.6 ± 1.8	6.6 ± 1.3	0.879*
RAL (mm, mean ± SD)	9.6 ± 2.0	10.0 ± 1.8	0.640*
Bone sounding (mm, mean ± SD)	11.8 ± 1.9	11.9 ± 2.0	0.867*
RGR (mm, mean ± SD)	2.9 ± 1.3	3.3 ± 1.1	0.352*
Measurements at defect sites			
Defect depth (mm, mean ± SD)	6.5 ± 1.6	5.6 ± 1.8	0.117*
Defect width (mm, mean ± SD)	3.4 ± 0.7	3.2 ± 0.7	0.312*

EMD: enamel matrix derivate; NHA: nanocrystalline hydroxyapatite; PPD: probing pocket depths; RAL: relative attachment level; RGR: relative gingival recession.

*Unpaired *t*-test, ^†^ *χ* ^2^ test, ^‡^Fisher's exact test.

**Table 2:** Clinical outcomes at 6 and 12 months.

Variable	Baseline (BS)	6 months	12 months	Difference (after 6 m)	*P* value* (Bs–6 m)	Difference (after 12 m)	*P* value* (Bs–12 m)	*P* value* (6 m–12 m)
Bone level (mm, mean ± SD)								
NHA group	11.8 ± 1.9	10.1 ± 2.1	10.1 ± 2.0	1.7 ± 1.8	0.001	1.6 ± 1.2	<0.001	0.886
EMD group	11.9 ± 2.0	10.2 ± 1.8	10.2 ± 1.8	1.8 ± 1.3	<0.001	1.6 ± 1.3	<0.001	0.919
*P* value^†^	0.867	0.836	0.867	0.958		1.000		
RAL (mm, mean ± SD)								
NHA group	9.6 ± 2.0	8.0 ± 2.3	8.1 ± 2.4	1.5 ± 2.0	0.004	1.4 ± 1.8	0.003	0.802
EMD group	9.8 ± 1.8	7.8 ± 1.7	7.7 ± 1.6	2.0 ± 1.6	<0.001	2.1 ± 1.6	<0.001	0.635
*P* value^†^	0.640	0.783	0.528	0.427		0.211		
PPD (mm, mean ± SD)								
NHA group	6.6 ± 1.8	3.9 ± 1.2	4.1 ± 1.7	2.7 ± 1.8	<0.001	2.6 ± 1.8	<0.001	0.706
EMD group	6.6 ± 1.3	3.4 ± 1.2	3.4 ± 1.1	3.2 ± 1.6	<0.001	3.2 ± 1.8	<0.001	0.936
*P* value^†^	0.879	0.191	0.154	0.425		0.312		
RGR (mm, mean ± SD)								
NHA group	2.9 ± 1.3	4.1 ± 1.3	4.1 ± 1.7	1.2 ± 1.2	0.001	1.1 ± 1.1	0.001	0.891
EMD group	3.3 ± 1.1	4.4 ± 1.2	4.3 ± 1.3	1.2 ± 1.1	<0.001	1.2 ± 1.2	0.001	0.552
*P* value^†^	0.352	0.518	0.596	0.946		0.785		

EMD: enamel matrix derivative; NHA: nanocrystalline hydroxyapatite; PPD: probing pocket depths; RAL: relative attachment level; RGR: relative gingival recession; *paired *t-*test; ^†^unpaired *t-*test. Mean differences are calculated as baseline 6–months, baseline–12 months, and 6–12 months.

**Table 3:** Full-mouth plaque scores.

Variable	Baseline	3 months	6 months	9 months	12 months	*P* value* (Bs–3 m)	*P* value* (Bs–6 m)	*P* value* (Bs–9 m)	*P* value* (Bs–12 m)
Plaque (%, mean ± SD)									
NHA group	13.2 ± 10.6	19.5 ± 11.0	28.4 ± 22.0	24.9 ± 17.2	21.3 ± 14.5	0.040	0.014	0.015	0.035
EMD group	9.6 ± 8.6	20.0 ± 14.0	24.7 ± 17.8	20.5 ± 16.1	19.1 ± 15.1	0.001	0.003	0.007	0.005
*P *value^†^	0.257	0.909	0.577	0.423	0.648				

*Paired* t-*test; ^†^unpaired *t-*test.
